# The Reference Genome Sequence of *Saccharomyces cerevisiae*: Then and Now

**DOI:** 10.1534/g3.113.008995

**Published:** 2013-12-27

**Authors:** Stacia R. Engel, Fred S. Dietrich, Dianna G. Fisk, Gail Binkley, Rama Balakrishnan, Maria C. Costanzo, Selina S. Dwight, Benjamin C. Hitz, Kalpana Karra, Robert S. Nash, Shuai Weng, Edith D. Wong, Paul Lloyd, Marek S. Skrzypek, Stuart R. Miyasato, Matt Simison, J. Michael Cherry

**Affiliations:** * Department of Genetics, Stanford University, Stanford, California 94305; † Department of Molecular Genetics and Microbiology, Duke University, Durham, North Carolina 27710

**Keywords:** *Saccharomyces cerevisiae*, model organism, reference sequence, genome release, S288C

## Abstract

The genome of the budding yeast *Saccharomyces cerevisiae* was the first completely sequenced from a eukaryote. It was released in 1996 as the work of a worldwide effort of hundreds of researchers. In the time since, the yeast genome has been intensively studied by geneticists, molecular biologists, and computational scientists all over the world. Maintenance and annotation of the genome sequence have long been provided by the *Saccharomyces* Genome Database, one of the original model organism databases. To deepen our understanding of the eukaryotic genome, the *S. cerevisiae* strain S288C reference genome sequence was updated recently in its first major update since 1996. The new version, called “S288C 2010,” was determined from a single yeast colony using modern sequencing technologies and serves as the anchor for further innovations in yeast genomic science.

Research regarding the genetics of yeast began in earnest during the 1930s and 1940s, with the pioneering work of Øjvind Winge (Szybalski 2001) and the work of Carl Lindegren (Lindegren 1949). The 16 chromosomes of *Saccharomyces cerevisiae* comprise the first completely finished eukaryotic genome and were sequenced in the early 1990s by an international consortium of researchers from 19 countries working in 94 laboratories using several different sequencing methods and technologies (Goffeau *et al.* 1996). The genome sequence is that of strain background S288C, and the strains used for the sequencing were predominantly AB972 (ATCC 76269) and FY1679 (ATCC 96604), two strains isogenic with S288C. Some sections of chromosome III were sequenced from XJ24-4a, A364A (ATCC 204626), and DC5 (ATCC 64665), and a small portion of chromosome XIV was taken from strain A364A (Table 1).

**Table 1:** Original *Saccharomyces cerevisiae* genome sequencing project

Chromosome	Length (bp)	Sequencing Coordinator	Original Strain*^a^*	Sequencing Methodology	Initial Release	Updated Versions
I	230,218	H. Bussey, Canada	AB972	Manual, automated	April 1995	11
II	813,184	H. Feldmann, Germany	S288C	Diverse methods in collaborating laboratories	December 1994	13
III	316,620	S. Oliver, England	XJ24-24a, AB972, A364A, DC5	Diverse methods in collaborating laboratories	May 1992	5
IV	1,531,933	C. Jacq, France	AB972, FY1679	Automated	May 1997	10
		B. Barrell, England				
		M. Johnston, United States				
		R. Davis, United States				
V	576,874	R. Davis, United States	AB972	Automated	May 1997	1
VI	270,161	Y. Murakami, Japan	AB972	Automated shotgun, primer walking	July 1995	3
VII	1,090,940	H. Tettelin, Belgium	FY1679	Diverse methods in collaborating laboratories	May 1997	9
VIII	562,643	M. Johnston, United States	AB972	Diverse methods in collaborating laboratories	September 1994	4
IX	439,888	B. Barrell, England	AB972	Shotgun, primer walking	May 1997	0
X	745,751	F. Galibert, France	FY1679	Diverse methods in collaborating laboratories	September 1996	10
XI	666,816	B. Dujon, France	FY1679	Diverse methods in collaborating laboratories	June 1994	9
XII	1,078,177	J. Hoheisel, Germany	AB972	Diverse methods in collaborating laboratories	May 1997	4
		M. Johnston, United States				
XIII	924,431	B. Barrell, England	AB972	Automated shotgun, primer walking	May 1997	1
XIV	784,333	P. Philippsen, Switzerland	FY1679, A364A	Diverse methods in collaborating laboratories	April 1996	8
XV	1,091,291	B. Dujon, France	FY1679	Manual, automated, shotgun, primer walking	May 1997	5
XVI	948,066	A. Goffeau, Belgium	AB972	Automated shotgun, primer walking	May 1997	2
		H. Bussey, Canada				
		R. Davis, United States				
		B. Barrell, England				
		M. Johnston, United States				

The original *Saccharomyces cerevisiae* genome sequencing project was a worldwide collaboration and chromosome sequences were subsequently updated independently numerous times before the recent major genome update.

a All strains are derived from S288C.

Here, we recount the genealogical history of S288C and the key derivative strains AB972 and FY1679. We also discuss the early *S. cerevisiae* sequencing efforts of the 1990s. Finally, we describe the resequencing and update of the *S. cerevisiae* reference genome.

## Materials and Methods

### Provenance of S288C

**Figure 1:**
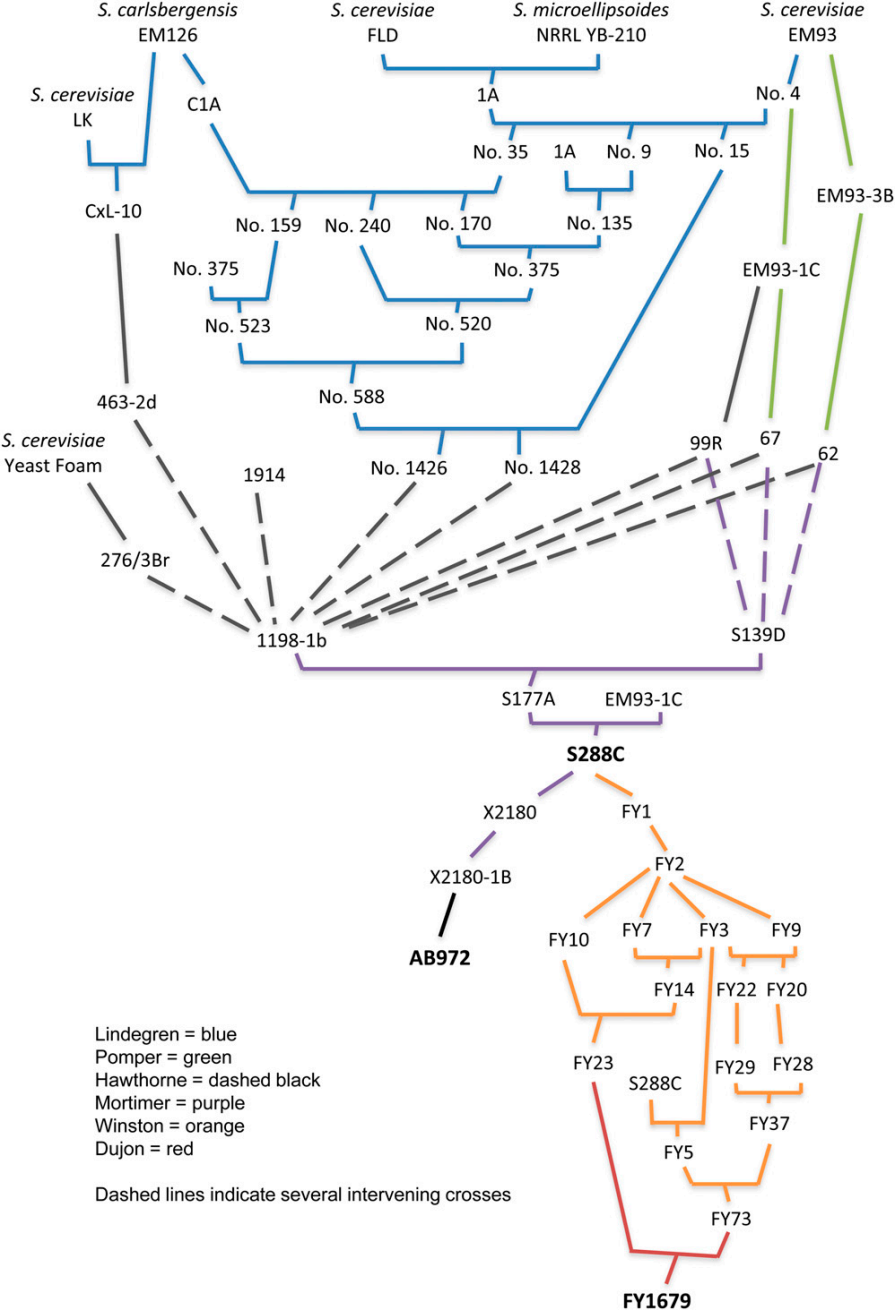
Genealogy of *Saccharomyces cerevisiae* strains S288C, AB972, and FY1679.

S288C is a common *gal2* mutant haploid laboratory strain with a long history of use in genetic and molecular biology studies. S288C has a complex genealogy; it is a contrived strain produced through numerous deliberate crosses, first by Carl Lindegren, and in later years by Robert Mortimer (Figure 1). Almost 90% of the S288C gene pool is from strain EM93, isolated by Emil Mrak in 1938 from a rotting fig collected outside the town of Merced in California’s Central Valley (Mortimer and Johnston 1986). Lindegren obtained Mrak’s EM93 for use in a laborious project to develop fertile breeding stocks for his genetic studies concerning the fermentation of different carbohydrates (Lindegren 1949). Lindegren obtained from L. J. Wickerham a culture of *Saccharomyces microellipsoides* (NRRL YB-210), a diploid-weak galactose and melibiose fermenter, but a nonfermenter of maltose. Wickerham had isolated NRRL YB-210 from a rotting banana that had been collected in Costa Rica in 1942 (C. Kurtzman, personal communication). Lindegren made a hybrid between haploid derivatives of NRRL YB-210 and his own isolate of FLD, a strong galactose and maltose fermenter that Lindegren referred to as a “standard legitimate diploid strain of commercial baking yeast” (Lindegren 1949). The hybrid between the NRRL YB-210 and FLD haploid derivatives produced only one viable ascospore (“1A”), which was completely incapable of fermenting galactose (Figure 1). Lindegren shared his yeast strains widely with other researchers. Seymour Pomper obtained from Lindegren an alpha mating–type haploid segregant of EM93 (by then renamed EM93-1C) as well as an “a” mating–type segregant of EM93 (EM93-3B). Pomper called these strains by the numbers 67 and 62 (Pomper 1952; Pomper and Burkholder 1949; Mortimer and Johnston 1986). Reaume and Tatum also obtained through Lindegren an alpha mating–type derivative of Mrak’s EM93 (EM93-1C) and used it in studies of spontaneous and induced nutritional deficiencies (Reaume and Tatum 1949). Mortimer obtained from Reaume strain 99R, an “adenineless” derivative of EM93-1C, and used 99R in his own studies of yeast genetics and isolation of the strain S288C.

Mortimer developed S288C to be used as a parental strain for the isolation of biochemical mutants. The immediate parents of S288C are EM93-1C (obtained by Cornelius Tobias from Lindegren and renamed SC7) and S177A, an EM93 derivative. One parent of S177A was S139D, which was derived by Mortimer entirely from Mrak’s EM93 via Reaume’s strain 99R and Pomper’s strains 67 and 62 (Mortimer and Johnston 1986). The other parent of S177A was 1198-1b, derived by Donald Hawthorne for genetic studies of the fermentation of sucrose, maltose, galactose, and alpha-methylglucoside (Hawthorne 1956). Hawthorne isolated 1198-1b through many crosses of a wide variety of strains, including Reaume’s strain 99R, Pomper’s strains 67 and 62, Lindegren’s 1426 and 1428, Ephrussi’s 276/3Br, which was a derivative of commercial baking strain Yeast Foam (Ephrussi *et al.* 1949), Lindegren’s 1914, and Roman and Douglas’s 463-2d, which was derived from Lindegren’s diploid CxL-10. The strain CxL-10 was derived by Lindegren from a cross between Mrak’s *Saccharomyces carlsbergensis* EM126 (a melibiose fermenter isolated from a rotting fig) and a commercial baking yeast called LK. The strain 1914 was deduced by Mortimer to have been isolated by Lindegren from crosses with the same set of strains that produced his 1426 and 1428 (Figure 1) (Lindegren 1949; Mortimer and Johnston 1986). It is no accident that S288C is widely used in laboratory settings. Mortimer bred it to be nonflocculent and to have only minimal nutrient requirements, including biotin, nitrogen, glucose, and various salts and trace elements (Mortimer and Johnston 1986).

### The early maps

All the crosses performed in the previous decades by Lindegren, Hawthorne, and others to investigate inheritance of mating type, nutritional requirements, metal resistance, and the fermentation of various sugars had ultimately led to the isolation of *Saccharomyces* strain S288C, but they also enabled construction of the first genetic and physical yeast chromosome maps. Lindegren published the first genetic maps of four chromosomes (Lindegren 1949). Lindegren and Lindegren (1951) presented maps of five chromosomes and introduced the Roman numeral system of yeast chromosome designations still in use today. In 1959, Lindegren and colleagues published more extensive maps for *Saccharomyces*, including nine chromosome arms with centromeres and one syntenic group without a centromere, stating that the haploid number of chromosomes was “at least seven” (Lindegren *et al.* 1959). A year later, Hawthorne and Mortimer (1960) published their first map, which included 10 chromosomes and maintained the Roman numeral designations of the Lindegrens from the map published in 1951. Mortimer and Schild published the first comprehensive genetic map of *S*. *cerevisiae* in 1980, which included 17 chromosomes and discussed the possible existence of an eighteenth (Mortimer and Schild 1980). Klapholz and Esposito (1982) soon showed that “chromosome XVII” was actually the left arm of chromosome XIV. Soon thereafter, Kuroiwa *et al.* (1984) used fluorescent staining of meiotic nuclei to demonstrate the presence of 16 chromosomes. By the publication of the 11^th^ edition of Mortimer’s map, the *S. cerevisiae* haploid chromosome number of 16 had become established and accepted (Mortimer *et al.* 1992).

### The original genome project

Like Lindegren, and in keeping with the collegial atmosphere that characterizes the yeast research community, Mortimer shared his S288C strain with other researchers. Fred Winston and colleagues (Winston *et al.* 1995) used gene replacement to develop a set of yeast strains isogenic to S288C but repaired for *GAL2*, which also contained nonreverting mutations in several genes commonly used for selection in the laboratory environment (*URA3*, *TRP1*, *LYS2*, *LEU2*, *HIS3*). Winston shared derivatives of this set, FY23 (mating type “a”) and FY73 (mating type “alpha”), with Bernard Dujon, who mated the strains to make the diploid FY1679, which was used in the original genome sequencing project (Figure 1) (Thierry *et al.* 1990). A cosmid library made from FY1679 was used for sequencing chromosomes VII, X, XI, XIV, and XV (Table 1) (Tettelin *et al.* 1997; Galibert *et al.* 1996; Dujon *et al.* 1994; Philippsen *et al.* 1997; Dujon *et al.* 1997). FY1679 was also used for sequencing the mitochondrial DNA, which was not part of the nuclear genome project and was determined separately (Foury *et al.* 1998). AB972 (Link and Olson 1991) is an ethidium bromide–induced *ρ°* derivative of Mortimer’s X2180-1B (obtained via Elizabeth Jones), itself a haploid derivative of strain X2180, which was made by self-diploidization of S288C (Figure 1) (Olson *et al.* 1986; Mortimer and Johnston 1986; Riles *et al.* 1993). AB972 was used in the original sequencing project as source DNA for chromosomes I, III, IV, V, VI, VIII, IX, XII, XIII, and XVI (Table 1) (Bussey *et al.* 1995; Oliver *et al.* 1992; Jacq *et al.* 1997; Dietrich *et al.* 1997; Murakami *et al.* 1995; Johnston *et al.* 1994; Churcher *et al.* 1997; Johnston *et al.* 1997; Bowman *et al.* 1997; Bussey *et al.* 1997). Portions of chromosome III were also taken from strains XJ24-24a, A364A, and DC5 (Oliver *et al.* 1992). Chromosome II was sequenced directly from strain S288C (Feldmann *et al*. 1994).

The sequencing project was launched in 1989, and it initially focused on chromosome III, which was chosen because plasmid and phage DNA libraries, as well as a physical map, were already available (Goffeau and Vassarotti 1991; Dujon 1996). Chromosome III was divided into contiguous overlapping fragments ∼10 kbp in length and distributed to 35 laboratories in 10 European countries (Vassarotti and Goffeau 1992). Each laboratory was allowed to apply sequencing strategies and methods of its own choosing, as long as they adhered to standards agreed on by the consortium (Goffeau and Vassarotti 1991). In the early years of the project, final sequences for the first completed chromosomes were deposited into the data library at the Martinsried Institute for Protein Sequences (MIPS) under the direction of H. Werner Mewes. MIPS provided initial sequence data coordination, warehousing, and analysis (Dujon 1992). As the project progressed, sequences for other chromosomes were deposited at the DNA DataBank of Japan (DDBJ), the European Molecular Biology Laboratory (EMBL), and GenBank, the National Institutes of Health (NIH) sequence database. The original annotation and maintenance of the chromosomal sequences were provided by each chromosome sequencing group (Vassarotti *et al.* 1995), MIPS, and SGD. In the early to mid 2000s, the data warehousing, annotation, and maintenance duties were assumed completely by SGD, which has participated in the annotation and maintenance of the genome sequence for the past 20 years.

The genome project had identified approximately 6000 protein-coding genes, many of unknown function (Goffeau *et al.* 1996); during this process, the researchers involved recognized the necessity for a stable systematic nomenclature. Through a series of consortium meetings in the late 1980s and early 1990s, members devised and revised the system currently in use today. Open reading frames (ORFs) were initially labeled with “Y” for yeast, alphabetical letters for chromosome (such as C for chromosome III), labeled with “L” or “R” for the left or right chromosome arm, and labeled with sequential numbers indicating their order within the specific plasmid or cosmid clone used for sequencing (B. Dujon, personal communication). This system allowed the numbering for different portions of chromosomes sequenced in different laboratories to be determined independently. The first published uses of this systematic nomenclature were from Thierry *et al.* (1990), who presented the sequence of an 8.2-kb segment of chromosome III with names starting at YCR521, and from Jacquier *et al.* (1992), who reported the sequence of a 10.7-kb section of chromosome XI with names starting at YKL500. Once the sequences of full chromosomes were being completed, it became clear that continuous numbering for each entire chromosome would be preferable. As a result, ORFs were renumbered starting with 1 at the centromere, then incrementing by +1 on each arm moving toward the telomere. The issue of strandedness for each ORF was subsequently addressed through the addition of the “W” or “C” suffix (for Watson or Crick). This systematic nomenclature was used in publication of the first complete sequence of a eukaryotic chromosome, that of yeast chromosome III (Oliver *et al.* 1992). The brevity and utilitarian nature of this nomenclature system have ensured its continued use and success.

### Surveys of the new genome sequence

Having the complete genome sequence available accelerated the progress of comprehensive surveys of different types of chromosomal elements that were already underway. Several different groups had been studying centromere structure (Hieter *et al.* 1985; Hegemann and Fleig 1993). Genomic mapping of replication origins (autonomously replicating sequences) had become an active area of investigation (Deshpande and Newlon 1992; Shirahige *et al.* 1993; Yang *et al.* 1999). Louis *et al.* (1994) were studying the mosaic structure of yeast telomeres. Moving forward, the complete sequence now enabled genomic surveys of different types of genes. Both Lowe and Eddy (1997) and Percudani *et al.* (1997) identified the entire set of transfer RNAs (tRNAs). Kim *et al.* (1998) identified hundreds of retrotransposon insertions. Planta and Mager (1998) determined the complete list of cytoplasmic ribosomal protein genes. Lowe and Eddy (1999) screened the genome for small nucleolar RNA (snoRNA) genes. In the 50 years after Lindgren first published manual drawings of four yeast chromosomes containing eight metabolic markers and one mating locus (Lindegren 1949), the yeast genome map had become almost fully populated.

### What is a reference genome?

Since its inception two decades ago, yeast genomics has been built around the single reference genome of S288C. The original idea was the production of a single consensus representative *S. cerevisiae* genome against which all other yeast sequences could be measured. The reference genome serves as the scaffold on which to hang other genomic sequences, and the foundation on which to build different types of genomic datasets. Whereas the first genome took years to complete, through the efforts of the large international consortium described, the sequences of dozens of genomes have been determined in the past several years (Engel and Cherry 2013). As sequencing has become more widespread, less novel, and, above all, less expensive, decoding entire genomes has become less daunting. New genomes now take only days to sequence to full and deep coverage and are assembled quickly, by individuals or small groups, through comparison to the reference, which is an invaluable guide for the annotation of newly sequenced genomes.

It is becoming increasingly clear that the genome of a species can contain a great deal of complexity and diversity. A reference genome can vary significantly from that of any individual strain or isolate and therefore serves as the anchor from which to explore the diversity of allele and gene complements and to explore how these differences contribute to metabolic and phenotypic variation. In the pharmaceutical industry, knowledge of the yeast reference genome helps drive the development of strains tailored to specific purposes, such as the production of biofuels, chemicals, and therapeutic drugs (Runguphan and Keasling 2013). In the beverage industry, it aids in the fermentation of beers, wines, and sakes with specific attributes, such as desired flavor profiles or reduced alcohol (Engel and Cherry 2013). We have seen the advantage afforded the yeast and genetics communities because of the early availability of an *S. cerevisiae* reference genome. The great facilitation of scientific discoveries and breakthroughs is without question (Botstein and Fink 2011).

### Maintenance of the genome annotation

The original genomic sequence and its annotation have been publicly available and tested by researchers around the world for the past 20 years. During that time, large numbers of corrections to the sequence and its annotation were proposed or published, and many of those were incorporated into the original reference genome sequence of 1996. New genes and other chromosomal features have been identified and added to the annotation, whereas others have been changed or deprecated (Fisk *et al.* 2006).

In the past several years, changes to the reference genome became less frequent as we moved toward a more stable and “correct” reference sequence. During the 5 yr spanning 2006–2010, 29 small sequence changes and 116 annotation updates were made. In addition, 576 new features were added to the genome annotation, including various ORFs, noncoding RNAs, mating cassette domains, autonomously replicating sequences (ARS), ARS consensus sequences (ACS), and 5′ untranslated region (UTR) introns. Clear descriptions of sequence and annotation changes for affected regions are available from the Locus History and Chromosome History pages of SGD (Table 4). SGD has always made new data available in a timely manner, such that before the recent major update of the entire genome, updates to individual chromosomes were made and released independently. As a result, between the original genome sequence and this new reference, SGD released 95 individual updated versions of the 16 nuclear chromosomes (Table 1). Whereas some chromosome sequences were never edited before now (*e.g.*, chromosome IX), others changed several times over the 15-year period. Chromosome III, which had been sequenced before any other chromosomes as a pilot project from DNA libraries prepared from four different S288C-derivative strains (AB972, XJ24-24a, A364A, DC5), was completely resequenced in the late 1990s from strain FY1679 by the laboratories of G. Volckaert and G. Valle, who submitted the sequence to GenBank/EMBL but did not otherwise publish the revision.

The latest *S. cerevisiae* version R64 genomic reference sequence (also known as S288C 2010) was determined in a single laboratory from a single colony of S288C-derivative strain AB972. This clone was from a stored isolate from the original AB972 strain used by Linda Riles to create the DNA libraries for some chromosomes in the original genome project (Table 1). Recent advances in the development of DNA sequence technologies have allowed the genome to be decoded from a single individual, in this case a single yeast colony, so that the reference genome truly is a single genome. In this article, we describe the sequence and annotation changes made to the *S. cerevisiae* reference genome in the first major update to the yeast genomic sequence.

The “S288C 2010” *S. cerevisiae* reference sequence version was determined from an individual AB972 yeast colony. Strain AB972 was obtained from M. Olson (Olson *et al.* 1986; Link and Olson 1991). Genomic DNA was isolated using standard protocols (Amberg *et al.* 2005). DNA was sheared and library construction was achieved with the Illumina TruSeq DNA Sample Prep kit. Illumina HiSequation 36-base sequencing was used. Data were generated as FASTQ files. Alignment and mapping of sequence reads to the previous version of the reference genome sequence (release R63.1.1, 2010-01-05) were accomplished using the Burrows-Wheeler Aligner (BWA) (Li and Durbin 2009). The resequencing covered only unique areas of the genome. Regions of repetitive sequence, including some microsatellites, transposable elements, telomeric regions, tRNA genes, and other miscellaneous repeats and GC-rich regions, together accounting for approximately 10% of the genome, were excluded from the analysis because sequence coverage was low or reads were of suboptimal quality. Using standard sequence quality scores, low-quality mismatches with the reference genome sequence version R63.1.1 were ignored. Only high-quality discrepancies were individually investigated through careful manual assembly and editing. The genome coordinates of each feature were updated using the LiftOver software tool available from UCSC Genome Bioinformatics (Hinrichs *et al.* 2006). Polymorphisms in coding regions were inspected manually to exclude a number of dubious calls and further refined by expert analysis to ensure the proper placement of start and stop codons. Sequence and annotation differences were checked against the published literature for any previous reports.

## Results

**Figure 2:**
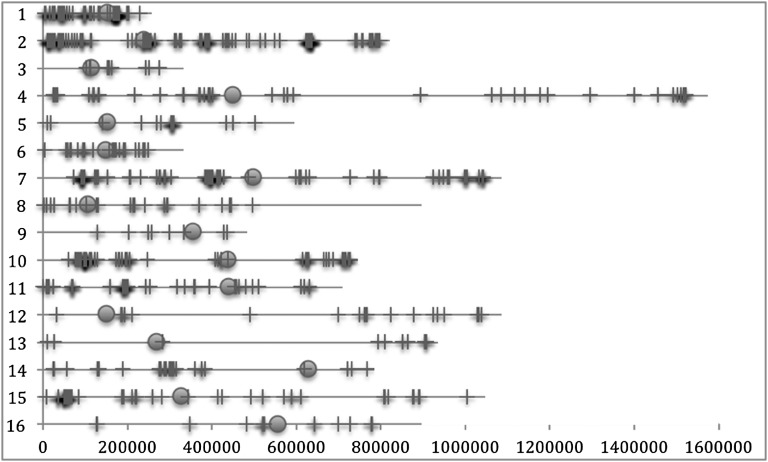
Chromosomal distribution of sequence changes. The sequences of all 16 nuclear chromosomes were updated, with changes between the previous genome version R63 and the current genome version R64 unevenly distributed throughout the genome. The X axis indicates chromosomal coordinates. Circles indicate centromeres.

We compared the new genome sequence to our previous version and corrected the sequence according to these results. The sequences of all 16 nuclear chromosomes were updated, with changes occurring in a nonrandom distribution (Figure 2). A number of coding sequences were changed, resulting in amino acid sequence changes to 194 proteins and silent changes in 42 ORFs (Supporting Information, Table S1). This represents approximately 3% of protein coding genes. Other updated features included one 5′ UTR intron, two ncRNAs, two tRNAs, 16 ARSs, one retrotransposon, one long terminal repeat (LTR), three telomeres, and 232 intergenic regions (Table 2). The largest sequence change was a 352-nucleotide insertion on chromosome XI in the intergenic region between ORFs *PMU1*/YKL128C and *MYO3*/YKL129C. Chromosome XI was originally sequenced from strain FY1679. It is unclear whether this difference represents real variation between strains AB972 and FY1679 or if it is an artifact of the construction or distribution of the cosmid library used for sequencing by the various participating laboratories (Dujon *et al.* 1994). Numbers of changed regions in each of the different chromosomes did not correlate with chromosome length (r = 0.253) or sequencing technology used. It is important to note that the quality of the original 1996 genome sequence was very high regardless of which sequencing technology (manual using Maxam-Gilbert or Sanger methods or automated using ABI sequencers) or assembly method (computational assembly or manual piecemeal integration of each cosmid sequence) was used. This is a testament to the care taken by the dozens of individuals working on each chromosome during the original genome sequencing project.

**Table 2:** Numerous features on the 16 nuclear chromosomes were updated in the latest genome release

**Chromosome**	Intergenic	ORF	Silent	Intron	5′ UTR Intron	ncRNA	tRNA	ARS	Retrotransposon	LTR	Telomere
I	17	17	2			1					
II	36	45	9	1	1			5			
III	6	4	1								
IV	23	15	1								
V	7	5						1			
VI	13	8	3	1		1	1				1
VII	30	23	6	1				2			
VIII	11	11	1	1				4			1
IX	4	2					1				
X	24	26	5					3	1	1	
XI	11	17	1	2							1
XII	11	9	3								
XIII	5	4	1								
XIV	9	15	2	1							
XV	19	15	6					1			
XVI	6	5	1								
**Total**	**232**	**221**	**42**	**7**	**1**	**2**	**2**	**16**	**1**	**1**	**3**

The sequences of various features on the 16 nuclear chromosomes were updated in the latest genome release R64.1.1. In addition to 194 altered protein sequences, 42 ORFs underwent silent coding changes. Other updated features included one 5′ UTR intron, two ncRNAs, two tRNAs, 16 ARSs, one retrotransposon, one LTR, three telomeres, and 232 intergenic regions.

The original 1996 reference genome sequence was determined primarily from AB972 and FY1679, both derivatives of S288C (Figure 1). The “S288C 2010” sequence reported here, determined from AB972, has been compared to a subsequently produced genome sequence of FY1679. There are approximately four SNPs per 100,000 bp between the “S288C 2010” reference and FY1679 (G. Song and B. Dunn, personal communication) demonstrating that these two strains are virtually indistinguishable.

### Genome versioning system

SGD has instituted a new versioning system, and this latest sequence update constitutes Genome Release 64.1.1 (released February 3, 2011, and still the latest version as of the writing of this article). In this nomenclature, the first number represents the reference sequence release, the second number represents the feature coordinate release within that sequence release, and the third number represents the annotation release. The reference sequence release number increments only when the reference sequence changes because of nucleotide insertion, deletion, or substitution. Moving forward, we anticipate such sequence change events will be exceedingly rare. The second number, the feature coordinate release number, will increment only when existing annotated features are altered (such as changing the translation start of an ORF) or when new genes or other chromosomal features are added. The last number, the annotation release, will increment when significant functional, Gene Ontology (GO), or phenotype information is added or updated. Although SGD biocurators typically add or update functional, GO, and phenotype information on a daily basis, once the versioning system is completely active, the annotation releases will be incremented once per week with the production of new files available from the SGD Downloads site (http://downloads.yeastgenome.org).

As stated, SGD released 95 total individual updated versions of the 16 nuclear chromosomes between the original release and this latest major update. Those chromosome sequence updates were released as independent events, although different chromosomes were sometimes updated on the same day. SGD has applied the new genome versioning system retroactively, such that chromosomal updates that were released simultaneously are now batched into a single genome version. The initial version is considered 1.1.1, and the 95 updated chromosome sequences correspond to Genome Releases 2.1.1 (released July 27, 1997) through 63.1.1 (released January 5, 2010) (Table 3). Of particular interest are SGD Genome Releases that correspond to genome versions used in the UCSC Genome Browser (http://genome.ucsc.edu/). UCSC release sacCer1 uses R27.1.1, sacCer2 uses R61.1.1, and sacCer3 matches the latest release, R64.1.1. SGD also provides LiftOver chain files between all previous releases and the current version of the S288C reference sequence, which allow researchers to convert data based on previous versions to current coordinates (for URL see Table 4).

**Table 3:** SGD genome versioning system

Genome Release	Date	Chromosome sequences updated
R1.1.1	1996-07-31	Initial release of 16 nuclear chromosomes
R2.1.1	1997-07-27	II, III, X, XIV
R3.1.1	1997-07-30	XII
R4.1.1	1997-08-11	XV
R5.1.1	1998-05-21	III
R6.1.1	1998-09-13	I, II
R7.1.1	1999-01-28	XIV
R8.1.1	1999-02-06	Mitochondrion*^a^*
R9.1.1	1999-02-10	IV
R10.1.1	1999-03-12	XI
R11.1.1	1999-04-22	II
R12.1.1	1999-04-26	II
R13.1.1	2000-01-21	VIII
R14.1.1	2000-03-16	V
R15.1.1	2000-04-21	IV
R16.1.1	2000-09-13	III
R17.1.1	2001-05-29	II, VI
R18.1.1	2001-05-31	VII, XI, XV
R19.1.1	2001-06-12	XII
R20.1.1	2001-06-29	X
R21.1.1	2002-12-19	I, IV
R22.1.1	2003-01-03	II, VII, X
R23.1.1	2003-01-09	VII, XI, XV
R24.1.1	2003-01-10	XI
R25.1.1	2003-09-26	VI
R26.1.1	2003-09-29	I, II, X, XVI
R27.1.1	2003-10-01	IV
R28.1.1	2003-12-15	I
R29.1.1	2004-01-12	II
R30.1.1	2004-01-14	I
R31.1.1	2004-01-23	II, VII
R32.1.1	2004-01-24	I
R33.1.1	2004-01-27	II
R34.1.1	2004-01-30	I, II
R35.1.1	2004-02-01	VIII
R36.1.1	2004-02-06	IV, VI, XII
R37.1.1	2004-02-13	IV, X, XI, XV
R38.1.1	2004-02-20	III, X, XIV
R39.1.1	2004-02-27	XIII
R40.1.1	2004-07-09	II
R41.1.1	2004-07-16	II, VII
R42.1.1	2004-07-23	I, IV, VII, XI, XIV, XVI
R43.1.1	2004-07-26	VIII
R44.1.1	2005-11-03	XIV
R45.1.1	2005-11-07	XIV
R46.1.1	2005-11-08	VIII
R47.1.1	2005-11-23	VII
R48.1.1	2005-12-02	VII, XI
R49.1.1	2005-12-16	XI
R50.1.1	2006-01-06	XV
R51.1.1	2006-01-13	III, XII
R52.1.1	2006-01-20	I, IV, X
R53.1.1	2006-04-14	IV
R54.1.1	2006-10-06	X
R55.1.1	2006-11-10	XIV
R56.1.1	2007-04-06	I
R57.1.1	2007-12-12	VII
R58.1.1	2008-03-05	I
R59.1.1	2008-06-03	XI
R60.1.1	2008-06-04	X
R61.1.1	2008-06-05	IV
R62.1.1	2009-02-18	X
R63.1.1	2010-01-05	XIV
R64.1.1	2011-02-03	All nuclear chromosomes

SGD has instituted a genome versioning system. There are a total of 95 individual updated versions of the 16 nuclear chromosomes between the original release and this latest major update (R64.1.1).

a The mitochondrial chromosome was not part of the original genome project and was determined separately (Foury *et al.* 1998).

**Table 4:** Some of the different data types that can be accessed at SGD

Type of Information	URL at SGD
Downloads site	http://downloads.yeastgenome.org/
DNA and protein sequences	http://downloads.yeastgenome.org/sequence
Dates of genome releases	http://downloads.yeastgenome.org/sequence/S288C_reference/dates_of_genome_releases.tab
Protein sequences updated in R64.1.1	http://www.yeastgenome.org/archive/ChangedProteins-2011.shtml
History of all sequence and annotation updates	http://www.yeastgenome.org/cgi-bin/chromosomeHistory.pl
History of sequence and annotation updates for specific loci	http://www.yeastgenome.org/cgi-bin/locusHistory.pl
All chromosome sequence changes	http://downloads.yeastgenome.org/sequence/S288C_reference/all_chromosome_sequence_changes.tab
LiftOver chain files	http://downloads.yeastgenome.org/sequence/S288C_reference/genome_releases/liftover/
Yeast strain genomes	http://downloads.yeastgenome.org/sequence/strains

## Discussion

With this comprehensive update in place, we anticipate very few sequence changes in the future. The increased quality of the new reference is such that we will handle all future reported differences as variations from this reference, rather than as sequencing “errors.” However, because a reference genome must take into consideration the best representation for that organism rather than simply the sequence of one individual, SGD will consider the possibility of accommodating small changes, if those changes increase the utility of the reference sequence. For example, we have allowed sequence updates that result in bringing two neighboring, nonfunctional ORFs into a single corrected reading frame to form a complete, functional coding region (such as *FLO8*/YER109C) (Liti *et al.* 1996). As new genomic sequences of other direct derivatives of S288C become available, differences between these additional S288C-derived genomic sequences from the reference will be detailed as alleles or as observed variation.

New technologies and approaches are now pushing *S. cerevisiae* annotation past the limits of a system based exclusively on a single reference sequence. Current sequencing methods have made possible the determination of the genomic sequences of hundreds of *S. cerevisiae* wild and laboratory strains. Comparative genomics of different sequences provides an expanded understanding of the full genetic constituent parts of a species and helps in the definition of conserved regions and in the identification of cryptic sequence features such as binding sites and noncoding RNAs. Different *S. cerevisiae* genomes vary not only in specific nucleotide sequence but also in their complements of genes; many genes are lost or gained as isolated populations adapt to their environment (Sliwa and Korona 2005; Lopez-Maury *et al.* 2008; Gordon *et al.* 2009; Lin and Li 2011). For example, the S288C reference sequence is missing several genes that are well-characterized in other strains, such as *XDH1* (xylitol dehydrogenase) (Wenger *et al.* 2010), *KHS1* (killer toxin) (Goto *et al.* 1991), *TAT3* (tyrosine transporter) (Omura *et al.* 2007), and *BIO1* (pimeloyl-CoA synthetase) (Hall and Dietrich 2007). The composition of several gene families also differs between strains; for instance, S288C includes only one (*SUC2*) of an eight-member invertase gene family (Carlson and Botstein 1983). SGD currently includes these genes in the database designated as “not in the systematic sequence of S288C” (Hirschman *et al.* 2006).

To address this variety in genome content, we are working toward the development of a virtual *S. cerevisiae* pan-genome that will contain all the genes found within all sequenced *S. cerevisiae* strains and wild isolates. The *S. cerevisiae* pan-genome contains hundreds of genes that are found in some strains but not in others. A pan-genome more accurately describes the full genetic complement of a species and will, in the future, provide a valuable resource for the annotation of newly determined budding yeast genomes and for the functional analysis and comparison of observed variation within *S. cerevisiae*.

We have also begun integrating into the database the complete genomic sequences of other *S. cerevisiae* strains (Engel and Cherry 2013). We currently provide precomputed protein and coding DNA alignments (ClustalW) for each ORF, as well as ORF-specific dendrograms, which depict the degree of similarity of that ORF sequence among the set of strains in which it was identified. Furthermore, we continue to associate information regarding sequence variation with functional effects and phenotypic variations (Engel *et al.* 2010). The genomes of the various strains have already been incorporated into the Basic Local Alignment Search Tool (BLAST) datasets, available for searching against genomic and coding DNA, as well as protein sequences. All the strain DNA and protein sequences are available for download so that researchers can perform their own analyses (http://downloads.yeastgenome.org/sequence). New tools are being developed that will provide access to this compendium of allelic and variation information and will allow any determined genomic sequence to be compared with the reference strain, as well as with the sequences of other widely used and commonly studied *S. cerevisiae* strains.

In their review of the publication of the complete genomic sequence of *S. cerevisiae* two decades ago, Clayton *et al.* (1997) lauded it as an enormous achievement and turned our gaze toward the future. Now, with a modern stable *S. cerevisiae* reference genome in place and a fresh appreciation of the inherent differences between strains, the next trend in yeast genomic science will focus on the elucidation and documentation of sequence variation and the biological and evolutionary consequences thereof. Yeast genomics has entered a new era and, once again, the greatest significance lies in the work that is yet to come.

### DNA accession numbers

The accession numbers for the 16 *S. cerevisiae* nuclear chromosomes and mitochondrial genome within the Reference Sequence (RefSeq) collection at the National Center for Biotechnology Information (NCBI) are as follows: NC_001133; NC_001134; NC_001135; NC_001136; NC_001137; NC_001138; NC_001139; NC_001140; NC_001141; NC_001142; NC_001143; NC_001144; NC_001145; NC_001146; NC_001147; NC_001148; and NC_001224.

## Supplementary Material

Supporting Information
