# Supplementary material for: The Reference Genome Sequence of Saccharomyces cerevisiae: Then and Now
Source: G3 (Bethesda). 2013 Dec 27;4(3):389–98. doi: 10.1534/g3.113.008995 (PMC3962479; doi:10.1534/g3.113.008995)
Supplement: Supporting Information [file supp_4_3_389__index.html]

The Reference Genome Sequence of Saccharomyces cerevisiae: Then and Now — Supporting Information 

# The Reference Genome Sequence of *Saccharomyces cerevisiae*: Then and Now

## Supporting Information for Engel *et al.*, 2014

**Files in this Data Supplement:**

- Table S1 - The sequences of all 16 nuclear chromosomes were updated in the latest genome release R64.1.1. In addition to 194 altered protein sequences, 40 ORFs underwent silent coding changes. Other updated features included one 5’ UTR intron, 2 ncRNAs, 2 tRNAs, 13 ARSs, 1 retrotransposon, 1 LTR, 3 telomeres, and 232 intergenic regions. Coordinates listed are chromosomal, and sequences are Watson strand. (.xls, 196 KB)
